# Retinal assessment in 143 patients with white matter lesions: the potential of OCTA as an evaluation tool

**DOI:** 10.3389/fneur.2025.1421232

**Published:** 2025-02-06

**Authors:** Dong Zhang, Xueying Jiang, Yan Zhang, Jingcui Qin

**Affiliations:** ^1^Department of Neurology, The Affiliated Xuzhou Municipal Hospital of Xuzhou Medical University, Xuzhou First People’s Hospital, Xuzhou, China; ^2^Department of Ophthalmology, The Affiliated Xuzhou Municipal Hospital of Xuzhou Medical University, Xuzhou First People’s Hospital, Xuzhou, China; ^3^Department of Geriatrics, The Affiliated Xuzhou Municipal Hospital of Xuzhou Medical University, Xuzhou First People’s Hospital, Xuzhou, China

**Keywords:** white matter, retina, fundus oculi, cognitive dysfunction, cerebral small vessel diseases

## Abstract

**Background:**

The retina is a simple window to reflect the changes of brain nerves. The aim of this study was to investigate the association between retinal status and white matter lesions (WMLs) in 143 patients aged 50–80 years, utilizing Optical Coherence Tomography Angiography (OCTA) and the Fazekas Visual Scale for assessment.

**Methods:**

A total of 143 subjects underwent MRI scanning to assess the degree of WMLs using the Fazekas scale. Retinal imaging was conducted utilizing OCTA. Multivariate logistic regression analysis was employed to determine the odds ratios and 95% confidence intervals associated with the Fazekas score for each factor. The relationship between cognitive function and various OCTA parameters was assessed using quadratic and cubic simulation curve models. Data following a normal distribution are presented as means, while non-normally distributed data are reported as medians. Qualitative data are expressed as percentages.

**Results:**

As Fazekas grades increased, ILM-IPL thickness (OR = 3.267, OR = 8.436), vessel density in the parafoveal region of the macula, retinal vessel densities (OR = 2.058, OR = 2.363), and RNFL thickness significantly reduced (*p* < 0.05). With increasing Fazekas scores, the bilateral foveal avascular zone showed a significant increase (OR = 0.362, OR = 0.458) (*p* < 0.05). Retinal thickness and vascular density were negatively correlated with WMLs, and positively correlated with cognitive function (*p* < 0.05).

**Conclusion:**

The severity of WMLs increases as retinal thickness and vascular density decrease. OCTA examination has a degree of role in screening for WMLs caused by cerebral microvascular disease. Its effectiveness in screening early asymptomatic individuals or those with mild cognitive impairment is somewhat limited.

## 1 Introduction

White matter lesions (WMLs) are frequently observed during cranial examinations of middle-aged and elderly individuals (1). It has been previously demonstrated that increased severity of WMLs is associated with a higher incidence of ischemic stroke (2). Numerous studies have also indicated that patients with more severe WMLs tend to have poorer prognoses following ischemic stroke compared to those without such lesions (3), and they experience greater degrees of cognitive impairment (4). WMLs may be caused by cerebral small vessel disease and reduced cerebral white matter blood flow (5). At present, the cause of WMLs is often attributed to chronic small vessel diseases. Some studies have found that reduced cerebral perfusion may cause bilateral cortical ischemia and hypoxia, thus leading to microcirculation disorders and worsening neurodegeneration (6). Secondary cortical damage occurs because the connections between the white matter fibers are damaged (7). However, in addition to risk factors, including age and hypertension, the severity of retinal microvascular abnormalities has been associated with the occurrence and development of lacunar infarcts (8) and WMLs (9), as reported in multiple studies. A reduction in the number of retinal microarterioles and microvenules, as well as a decrease in the thickness of the inner layer of the retina, are significantly associated with impaired cognitive abilities, lower volumes of gray and white matter, and compromised white matter network structures (10).

The retinal and cerebral blood vessels have the same embryonic origin and similar anatomical and pathophysiological characteristics (11). During embryonic development, the retina and optic nerve are direct extensions of the brain. In terms of the microvascular system, retinal arterioles and venules have similar features to cerebral small blood vessels (12), including non-anastomotic terminal arterioles, barrier function, and autoregulation. Furthermore, they have a relatively low flow rate and a high oxygen extraction system. The anatomical relationship between their blood supplies and the two vascular networks have similar vascular regulatory processes. Therefore, retinal vessels can reflect changes in cerebral vessels (13).

Furthermore, the retinal nerve fiber layer (RNFL) is the innermost layer of the retina, composed of axons of retinal ganglion cells. It connects the retinal nerve to the lateral geniculate nucleus and synapses with the visual cortex, thus serving as a projection of the central nervous system. Therefore, the retinal vessels, as the only terminal microvessels that can be directly observed *in vivo*, share similar embryological, anatomical, and physiological characteristics with cerebral small vessels. They provide a practical visual “window” for clinical investigations concerning alterations in cerebral microvessels (14).

The retina serves as a simple window for monitoring neurodegeneration in the brain (15). By evaluating the density and thickness of retinal vessels, neurodegenerative conditions within the skull can be indirectly identified using non-invasive methods. Optical coherence tomography angiography (OCTA) is an innovative technology that facilitates microcirculation imaging by processing optical coherence tomography (OCT) data (16). This technique enables the acquisition of high-resolution three-dimensional retinal vasculature images through non-invasive means. Compared to fundus angiography, OCTA offers enhanced safety, speed, and non-invasiveness. Moreover, it surpasses fundus photography in depicting certain features of retinal vascular pathologies, such as neovascularization, ischemia, and microaneurysms (17). In recent years, OCTA has emerged as a widely adopted research method for investigating retinal vascular changes. It provides a highly specific and sensitive, simple, reproducible, and non-invasive approach for studying cerebrovascular small vessel disease. Nevertheless, the majority of existing studies struggle to quantify the relationship between retinopathy and WMLs using precise metrics (18). These studies utilized OCTA to acquire and present fundus blood vessel images, drawing conclusions based solely on visual assessment of the differences in vascular density between the two groups as observed in the images (19). While several studies have gathered specific quantitative data via OCTA and provided positive conclusions regarding retinal blood vessels, a comprehensive and detailed analysis of intraretinal vascular parameters has not been fully conducted (20). Consequently, this study examined the variations in retinal thickness and vascular density among patients with varying degrees of white matter injury by utilizing multiple quantitative OCTA metrics.

Additionally, the relation and mechanism between retinal vascular changes and cognition in WMLs patients were explored. The objective is to identify new potential biomarkers (retinal vascular changes) for cognitive impairment in patients with WMLs or cerebral small vessel disease and provide a new perspective on their neurobiological mechanisms. We hypothesize that retinal thickness and vascular density may be negatively correlated with WMLs grading and positively correlated with cognitive function. Therefore, this study aimed to evaluate the retinal vessels using OCTA and changes in cerebral white matter using the Fazekas visual rating scale in 143 patients between 50 and 80 years.

## 2 Methods

### 2.1 Ethical statement

The Ethics Committee of the First People’s Hospital of Xuzhou approved this study (Approval number: xyyll[2023]029). All participants or their legal representatives duly signed informed consent forms. All participants underwent OCTA examination after signing informed consent forms.

### 2.2 Participants

This study included 143 patients with varying degrees of WMLs recruited from the Department of Neurology at the First People’s Hospital of Xuzhou between April 2022 and September 2023. Two neurologists screened suitable subjects from brain magnetic resonance imaging (MRI) findings of admitted or outpatient patients based on inclusion and exclusion criteria.

### 2.3 Inclusion criteria

The patients aged between 50 and 80 years, with the presence of clearly defined WMLs on cranial MRI, were included in this study. This criteria is applicable to all subjects.

### 2.4 Exclusion criteria

Exclusion criteria included the following: Presence of mental or consciousness disorders; cardioembolic stroke; recent acute ischemic stroke or history of severe stroke; congenital vascular diseases or cerebral artery occlusion; based on the history of toxic exposure and imaging findings, patients were diagnosed with brain injuries attributable to other etiologies, including but not limited to multiple sclerosis, hypoxia, or intoxication; history of contrast agent allergy or allergic reactions to medications more than twice in the past; cardiac dysfunction (the New York Heart Association Functional classification is greater than class II), hepatic dysfunction (glutamic pyruvic transaminase >80 U/L or glutamic oxal transaminase >80 U/L), or renal dysfunction (glomerular filtration rate < 90 ml/min); history of cranial trauma or surgery; high myopia or other eye diseases including glaucoma and retinal detachment, previous eye surgery or severe retinopathy due to hypertension or diabetes; presence of MRI contraindications, internal metal implants, or suffering from claustrophobia. This criteria is applicable to all subjects.

### 2.5 General data acquisition

General data included: gender, age, height, weight, previous history (hypertension, diabetes, coronary heart disease, etc.), smoking and drinking history, admission blood pressure, fasting blood glucose, blood lipids and other indicators.

### 2.6 OCTA examination

Binocular retinal imaging was performed using the RTVue XR Avanti spectral-domain OCT device (RTVue XR Avanti with AngioVue; Optovue Inc., Fremont, CA, USA). This spectral-domain OCT system utilizes a light source centered at 840 nm with a bandwidth of 45 nm and a scanning rate of 70,000 A-scans per second. For each subject, two consecutive B-scans were performed at the same location. Each B-scan comprised 304 A-scans to reduce motion artifacts caused by microsaccades and fixational eye movements. Information on retinal microvascular perfusion was extracted using the spectral amplitude-decorrelation angiography algorithm to track the movement of red blood cells by analyzing changes in OCT signal amplitude between consecutive cross-sectional scans. It provides quantitative vascular density, defined as the percentage of area occupied by vessels in the analyzed region on the OCTA image. The inclusion criteria required signal quality of the eye ≥5, signal strength index ≥40, and suboptimal sharpness without significant motion or shadow artifacts.

### 2.7 OCTA image acquisition and retinal quadrant division

Independent measurements by 2 experienced ophthalmologists. OCTA images of the macular area measuring 3.0 × 3.0 mm^2^ were obtained through scanning, with the OCT instrument automatically segmenting the retinal superficial capillary plexus (SCP) and deep capillary plexus (DCP). The SCP is located between 3 μm beneath the internal limiting membrane (ILM) and 15 μm below the inner plexiform layer (IPL), while the DCP is situated from 15 to 70 μm below the IPL. The software automatically divided the macular area into the foveal and parafoveal zones. The foveal zone was defined as a circular area with a diameter of 1 mm, and the parafoveal zone is a region between 1.0 and 3.0 mm in diameter. The parafoveal zone was further divided into four quadrants: superior (S), inferior (I), temporal (T), and nasal (N). Additionally, the software automatically calculated the area of the foveal avascular zone (FAZ), a few hundred microns-sized avascular area surrounded by capillaries in the fovea. The collected data included the vascular density of the retinal SCP and DCP in the parafoveal zone, FAZ area, and thickness of the ILM-IPL in the macula. For optic disc imaging, OCTA images measuring 4.5 × 4.5 mm^2^ were obtained. The software automatically segmented the radial peripapillary capillary (RPC) layer around the optic disc, which extended from the ILM to the outer boundary of the RNFL. The software fitted two concentric circles around the optic nerve head, with inner and outer circle diameters of 2.0 and 4.0 mm, respectively, defining the region around the optic disc as the area between these two circles. The peripapillary region was divided into eight sectors according to the Garway-Heath map: temporal superior (TS), superior temporal (ST), superior nasal (SN), nasal superior (NS), nasal inferior (NI), inferior nasal (IN), inferior temporal (IT), and temporal inferior (TI). Furthermore, the software automatically measured the RNFL thickness in the aforementioned regions. The collected data included vascular density (the ratio of the total vascular area to the overall area within a 3.0 × 3.0 mm^2^ region) and RNFL thickness of the RPC network in each sector around the optic disc.

### 2.8 OCTA image quality control

Subjects do not need to do any preparation before OCTA, such as mydriasis, and the subject can perform this examination at any time. The examination is non-invasive, so it does not require the use of any reagents or contrast agents. Ocular measurements were conducted bilaterally for all subjects. In this study, the relevant OCTA data were measured by two operators in the same sequence using a double-blind method. Then, a set of valid data was obtained by one of the operators in different order, and the repeatability of the two measurements within the operator was evaluated. All OCTA data is automatically captured and generated by RTVue XR Avanti (2018 version) software. Both operators perform standard operations according to the RTVue XR Avanti User manual.

### 2.9 MRI scan

MRI was performed using a superconductive magnetic resonance imaging system (GE Signa HDxt 3.0 T, USA) and standard orthogonal coils. During the examination, the patient was positioned supine with the head secured on a headrest. First, the following sequences were acquired: T1-weighted imaging with parameters TR of 1900 ms, TE of 20 ms, slice thickness of 5 mm, interslice gap of 1 mm, and a field of view (FOV) of 230 × 185 mm; T2-weighted imaging with parameters TR of 4,000 ms, TE of 104 ms, slice thickness of 5 mm, interslice gap of 1 mm, FOV of 230 × 209 mm; FLAIR imaging with parameters TR of 9,000 ms, TE of 120 ms, TI of 2,500 ms, slice thickness of 5 mm, and interslice gap of 1 mm. The images were reviewed by a radiologist who assisted in identifying and screening the areas of interest for WMLs. Patients selected on the basis of MRI results were re-screened by two neurologists according to the inclusion criteria.

### 2.10 WMLs analysis

Patients who agree to be enrolled are grouped according to the extent of their WMLs. The severity of WMLs was assessed using the Fazekas scale (21). The Fazekas scale is a scoring method that quantifies the severity of WMLs by observing the lesions on T2 Fluid-attenuated inversion recovery (FLAIR) images according to the formulated scoring rules. The Fazekas scale scores WMLs separately in periventricular and deep white matter regions (each section is 0–3 scores), and then the scores from both regions were combined to obtain a final score (total 0–6 scores). The scoring criteria for periventricular or deep white matter high signal intensity were as follows: 0 points indicated no lesions, 1 point indicated caps or pencil-thin lining lesions, 2 points indicated lesions forming smooth halos, and 3 points indicated irregular periventricular high signals extending into the deep white matter. The Fazekas scale categorizes the severity into grades from 0 to 3: Grade 0 indicates no lesions (total scores of 0, F0); Grade 1 (mild lesions, total scores of 1–2, F1); Grade 2 (moderate lesions, total scores of 3–4, F2); and Grade 3 (severe lesions, total scores of 5–6, F3). Patients were grouped according to their Fazekas grades. The subjects were grouped as mild based on the Fazekas grades: mild lesions (control group, F0–F1) were assigned Fazekas 0 and 1 grades, whereas severe lesions (experimental group, F2–F3) were assigned Fazekas 2 and 3 grades.

### 2.11 Cognitive function

All subjects underwent cognitive function assessment using the Montreal Cognitive Assessment (MoCA) and Mini-Mental State Examination (MMSE). The assessments were conducted by a professional psychosomatic medicine or neurology physician, strictly following the standard protocol and sequence of tests. Due to the low sensitivity of MMSE detection, it is difficult to distinguish patients with mild symptoms of cognitive dysfunction. This may lead to bias in the control analysis. Therefore, Moca score was mainly used for data analysis.

### 2.12 Statistical analysis

Normally distributed data are presented as mean ± standard deviation (*m* ± *sd*). Skewed distribution data are presented as the interquartile. Qualitative data are presented as percentages. Subsequently, we conducted a comparative analysis to examine the differences between the control group and the experimental group. The normality of the data was assessed using the Kolmogorov–Smirnov test. For non-normally distributed data, the Mann–Whitney *U* test was used to compare the two groups. Normally distributed data were compared using independent samples t-test. The categorical data were compared between groups using Pearson’s *χ^2^* test. Ordered multiple logistic regression analysis was used to calculate the odds ratios (OR) and 95% confidence intervals (95% CI) between Fazekas scores and OCTA parameters (such as RNFL, FAZ, blood vessel density, etc.). We adjusted for age, sex, hypertension, diabetes, and drinking in our multiple logistic regression analysis. The relationship between MoCA scores and OCTA parameters was modeled using quadratic and cubic curve simulations. Subsequently, the positive and negative relations between cognitive function and retinal vasculature were analyzed in greater depth based on the characteristics of each curve. Data analysis was performed using SPSS Statistics 26.0, with statistical significance set at *p* < 0.05. Given the extensive volume of OCTA data, only those datasets that hold greater significance for discussion were selected and presented in the table, such as the data with *p* > 0.05 or more pronounced intergroup differences. The remaining data are provided in Supplementary tables.

## 3 Results

### 3.1 General information

A total of 143 subjects were included in the study, including 70 males (49.0%) with a mean age of 64.6 years. Among the participants, 85 had Fazekas scores of 0 or 1, while 58 had scores of 2 or 3. Table 1 summarizes the demographic and background information of patients based on Fazekas scores. Significant differences were observed between groups for gender, hypertension, diabetes, and alcohol consumption. The experimental group exhibited a markedly higher prevalence of males with hypertension, diabetes, and a history of alcohol consumption (*p* < 0.05). In the OCTA data, only the vessel density in the right temporal-inferior quadrant did not show statistically significant differences between the two groups (*p* = 0.126). In contrast, all other OCTA indices demonstrated statistically significant differences between the groups (*p* < 0.05). Consistent with prior research, participants in the control group who exhibited higher Fazekas scores demonstrated significantly greater cognitive function scores compared to those in the experimental group with lower Fazekas scores (*p* < 0.001) (Table 1).

**Table 1:** Comparison of patient demographic data and background data based on Fazekas grades.

Variables	Control group (F0-F1)	Experimental group (F2-F3)	*p* value
*n*	85	58	
Age (Year, *m ± s*)	62 ± 7	68 ± 7	0.872^**^
Male (*n*, %)	33, 38.8	37, 63.8	0.003^***^
Duration of education exceeds 9 years (*n*, %)	51, 60	36, 62.1	0.803^***^
Hypertension (*n*, %)	43, 50.6	29, 87.9	0.002^***^
Dyslipidemia (*n*, %)	23, 27.1	13, 22.4	0.530^***^
Diabetes (*n*, %)	18, 21.2	21, 36.2	0.048^***^
Coronary heart disease (*n*, %)	13, 15.3	12, 20.7	0.404^***^
Fasting blood glucose (mmol/L, M, IQR)	5.54(4.90–6.36)	5.54(4.99–6.85)	0.535^*^
Smoke (*n*, %)	8, 9.4	12, 20.7	0.056^***^
Drinking (*n*, %)	6, 7.1	12, 20.7	0.016^***^
SBP on admission (mmHg, *m* ± *s*)	140.1 ± 19.7	143.7 ± 19.9	0.168^*^
DBP on admission (mmHg, *m* ± *s*)	84.8 ± 12.1	86.3 ± 13.2	0.477^**^
Cholesterol (mmol/L, M, IQR)	4.30 (3.60–5.20)	4.13 (3.45–5.10)	0.358^*^
Triglyceride (mmol/L, M, IQR)	1.17 (0.82–1.78)	1.27 (0.90–1.92)	0.711^*^
LDL-C (mmol/L, M, IQR)	2.69 (2.31–3.29)	2.53 (2.20–3.17)	0.261^*^
HDL-C (mmol/L, *m* ± *s*)	1.19 ± 0.29	1.13 ± 0.29	0.207^**^
ILM-IPL thickness OD (μm, M, IQR)	55 (52–59)	50 (47–52)	<0.001^*^
ILM-IPL thickness OS (μm, M, IQR)	56 (52–59)	50 (46–51)	<0.001^*^
ST RNFL thickness OD (μm, *m* ± *s*)	124.4 ± 13.7	112.0 ± 12.6	<0.001^**^
TI RNFL thickness OD (μm, *m* ± *s*)	84.2 ± 10.0	67.9 ± 9.7	<0.001^**^
IN RNFL thickness OS (μm, *m* ± *s*)	144.2 ± 12.9	129.6 ± 11.1	<0.001^**^
NS RNFL thickness OS (μm, *m* ± *s*)	92.3 ± 12.8	80.5 ± 10.0	<0.001^**^
ST vessel density OD (%, M, IQR)	56 (53–59)	53 (50–56)	<0.001^*^
TI vessel density OD (%, *m* ± *s*)	52.0 ± 4.2	50.8 ± 4.9	0.126^**^
IN vessel density OS (%, M, IQR)	51 (49–54)	50 (48–52)	0.019^*^
NS vessel density OS (%, M, IQR)	50 (48–52)	47 (45–48)	<0.001^*^
FAZ OD (mm^2^, *m* ± *s*)	0.268 ± 0.046	0.340 ± 0.051	<0.001^**^
FAZ OS (mm^2^, M, IQR)	0.274 (0.241–0.293)	0.347 (0.324–0.376)	<0.001^*^
SCP OD (%, M, IQR)	51.1 (49.6–53.7)	45.9 (42.2–49.5)	<0.001^*^
SCP OS (%, *m* ± *s*)	51.1 ± 3.5	46.0 ± 4.0	<0.001^**^
DCP OD (%, M, IQR)	51.4 (49.7–54.1)	47.3 (44.6–49.6)	<0.001^*^
DCP OS (%, *m* ± *s*)	51.5 ± 2.7	47.7 ± 3.2	<0.001^**^
MMSE (M, IQR)	29 (28–30)	27 (23–28)	<0.001^*^
MOCA (M, IQR)	25 (24–27)	20 (17–22)	<0.001^*^

SBP, Systolic blood pressure; DBP, Diastolic blood pressure; LDL, Low-density lipoprotein; HDL, High density lipoprotein; RNFL, Retinal nerve fiber layer; ILM, Internal limiting membrane; IPL, Inner plexiform layer; S, Superior; I, inferior; T, temporal; N, nasal; FAZ, Foveal avascular zone; SCP, Superficial capillary plexus; DCP, Deep capillary plexus; OD, Oculus dexter; OS, Oculus sinister; MoCA, Monterey Cognitive Assessment Scale; MMSE, Mini-Mental State Examination.

* Man-Whitney U test.

** Independent sample *t*-test.

*** Pearson Chi-square test.

### 3.2 Multivariate logistic analysis of WMLs severity and RNFL thickness, vessel density, retinal SCP, and DCP vessel density

In the multiple logistic regression analysis, the data from subjects with Fazekas grade of 3 was used as a reference. Among them, Fazekas grade of 3 were classified as the most severe patients and Fazekas grade of 0 was classified as normal subjects. Multivariate logistic analysis was conducted to explore the relationship between Fazekas grades and RNFL thickness, vessel density, FAZ area, retinal SCP, and DCP vessel density at different locations. As Fazekas grades increased, ILM-IPL thickness (F0: OR = 3.267, 95% CI = 2.026–5.269; OR = 8.436, 95% CI = 2.816–25.266), vessel density in the parafoveal region of the macula, both deep retinal vessel densities (F0: OR = 2.058, 95% CI = 1.487–2.850; OR = 2.363, 95% CI = 1.703–3.278) and superficial retinal vessel densities (F0: OR = 2.217, 95% CI = 1.584–3.103; OR = 2.059, 95% CI = 1.497–2.831), and RNFL thickness significantly reduced (all *p* < 0.05) (Table 2).

**Table 2:** Multiple logistic regression analysis between retinal vascular indexes and Fazekas grade.

Variables	Oculus Dexter (OD)		Oculus Sinister (OS)	
	OR (95% CI)	*p* value	OR(95%CI)	*p* value
ILM-IPL thickness(F3)	1.000	-	1.000	-
F2	1.503 (1.077–2.099)	0.017	3.967 (1.563–10.067)	0.004
F1	2.706 (1.705–4.293)	<0.001	7.792 (2.607–23.283)	<0.001
F0	3.267 (2.026–5.269)	<0.001	8.436 (2.816–25.266)	<0.001
*p* trend		<0.001		<0.001
ST RNFL thickness(F3)	1.000	-	1.000	-
F2	1.047 (0.964–1.137)	0.276	1.104 (1.016–1.200)	0.020
F1	1.093 (1.014–1.178)	0.020	1.120 (1.030–1.219)	0.008
F0	1.111 (1.025–1.204)	0.010	1.121 (1.025–1.226)	0.012
*p* trend		0.002		0.001
IN RNFL thickness(F3)	1.000	-	1.000	-
F2	1.095 (1.020–1.174)	0.012	1.366 (1.212–1.540)	<0.001
F1	1.138 (1.061–1.222)	<0.001	1.240 (1.112–1.384)	<0.001
F0	1.191 (1.105–1.284)	<0.001	1.131 (1.028–1.243)	0.011
*p* trend		<0.001		<0.001
TI vessel density(F3)	1.000	-	1.000	-
F2	1.295 (1.059–1.584)	0.004	1.251 (1.008–1.554)	0.043
F1	1.285 (1.069–1.545)	0.008	1.332 (1.084–1.636)	0.006
F0	1.355 (1.099–1.669)	0.012	1.265 (1.009–1.586)	0.042
*p* trend		0.002		0.068
NS vessel density(F3)	1.000	-	1.000	-
F2	1.378 (1.054–1.801)	0.019	2.345 (1.351–4.068)	0.002
F1	1.668 (1.252–2.221)	<0.001	4.204 (2.196–8.501)	<0.001
F0	2.083 (1.520–2.854)	<0.001	6.049 (3.057–11.970)	<0.001
*p* trend		<0.001		<0.001
FAZ(F3)	1.000	-	1.000	-
F2	0.619 (0.454–0.844)	0.002	0.759 (0.608–0.947)	0.014
F1	0.457 (0.320–0.654)	<0.001	0.490 (0.364–0.659)	<0.001
F0	0.362 (0.247–0.532)	<0.001	0.458 (0.337–0.623)	<0.001
*p* trend		<0.001		<0.001
SCP(F3)	1.000	-	1.000	-
F2	1.491 (1.113–1.997)	0.007	1.367 (1.034–1.807)	0.028
F1	1.871 (1.376–2.544)	<0.001	1.823 (1.353–2.455)	<0.001
F0	2.217 (1.584–3.103)	<0.001	2.059 (1.497–2.831)	<0.001
*p* trend		<0.001		<0.001
DCP(F3)	1.000	-	1.000	-
F2	1.378 (1.043–1.819)	0.024	1.738 (1.314–2.299)	<0.001
F1	1.803 (1.341–2.425)	<0.001	2.357 (1.724–3.222)	<0.001
F0	2.058 (1.487–2.850)	<0.001	2.363 (1.703–3.278)	<0.001
*p* trend		<0.001		<0.001

ILM, Internal limiting membrane; IPL, Inner plexiform layer; RNFL, Retinal nerve fiber layer; S, Superio; I, inferior; T, temporal; N, nasal; FAZ, Foveal avascular zone; SCP, Superficial capillary plexus; DCP, Deep capillary plexus; F0-3, Fazekas grades.

### 3.3 Multivariate logistic analysis of WMLs severity and FAZ area, ILM-IPL thickness

With increasing Fazekas grades, the bilateral FAZ showed a significant increase (F0: OR = 0.362, 95% CI = 0.247–0.532; OR = 0.458, 95% CI = 0.337–0.623) (*p* < 0.05). The OR values for Fazekas grade 0 data were significantly higher (*p* < 0.05) than those for Fazekas grade 3 data. In ILM-IPL thickness, the right eye thickness of Fazekas grade 0 subjects was 3.267 times higher than that of Fazekas grade 3 subjects (OR = 3.267, 95% CI = 2.026–5.269, *p* < 0.001). Whereas the left eye thickness of Fazekas grade 0 subjects was 8.436 times higher than that of Fazekas grade 3 subjects (OR = 8.436, 95% CI = 2.816–25.266, *p* < 0.001) (Table 2).

### 3.4 Quadratic and cubic simulated curve models between cognitive function and various OCTA parameters

**Figure 1:**
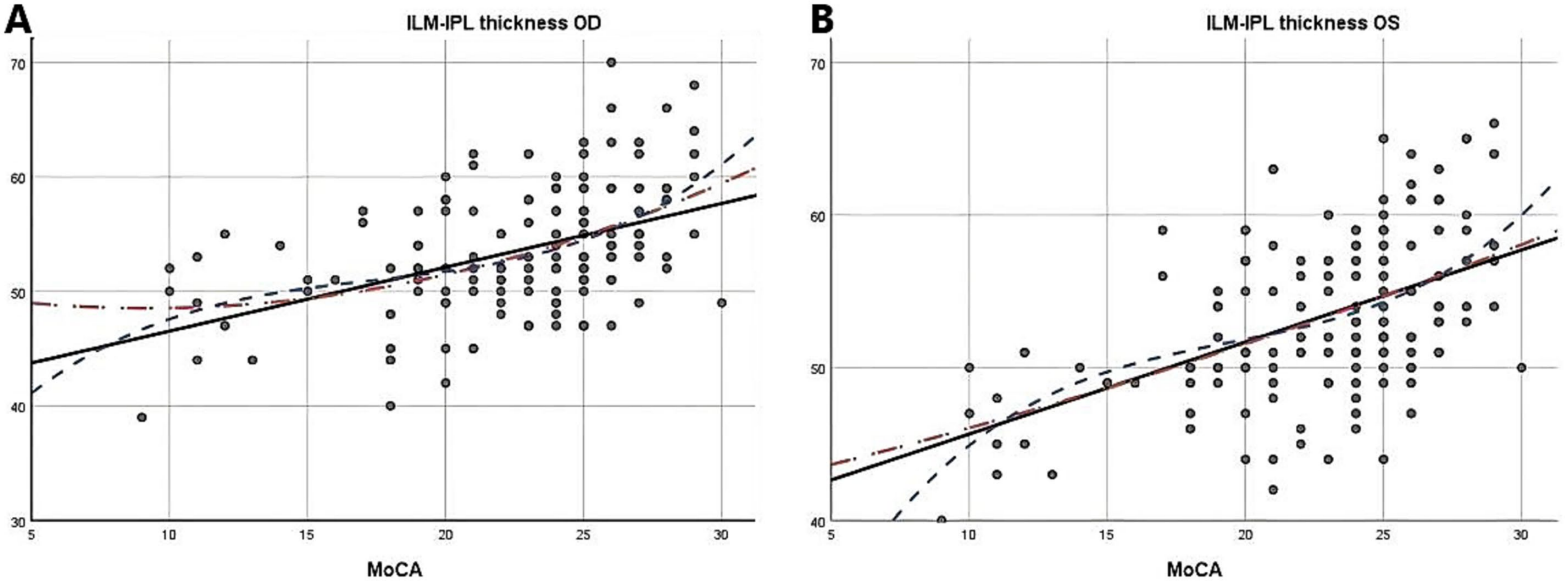
The relationship between Moca score and ILM-IPL thickness (SPSS Statistics 26 IBM); **A**: Moca score and right eye ILM-IPL thickness; **B**: Moca score and left eye ILM-IPL thickness; MoCA: Monterey Cognitive Assessment Scale; ILM: Internal limiting membrane; IPL: Inner plexiform layer.

**Figure 2:**
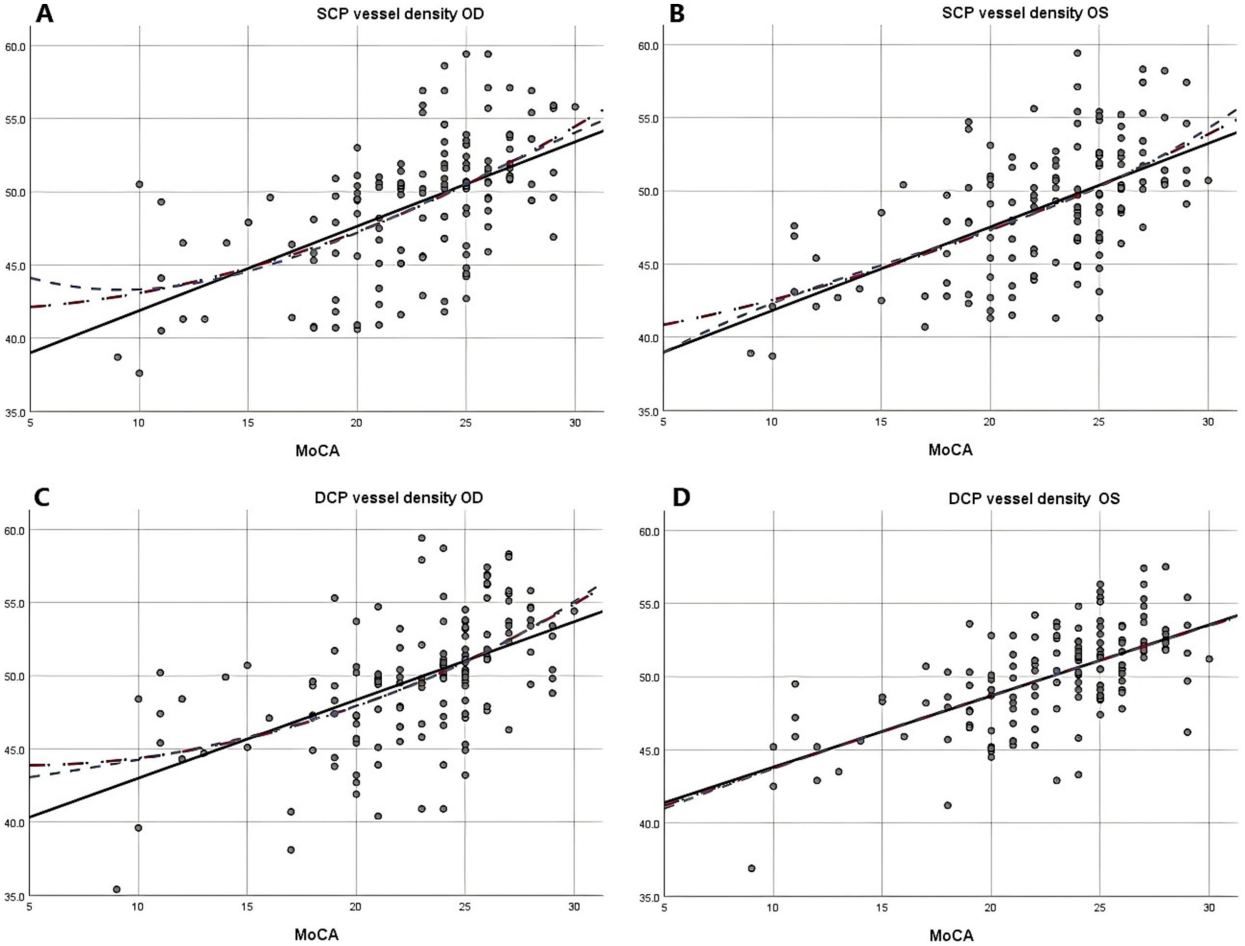
The relationship between Moca score and vascular density of SCP and DCP (SPSS Statistics 26 IBM); **A**: Moca score and right eye vascular density of SCP; **B**: Moca score and left eye vascular density of SCP; **C**: Moca score and right eye vascular density of DCP; **D**: Moca score and left eye vascular density of DCP; MoCA: Monterey Cognitive Assessment Scale; SCP: Superficial capillary plexus; DCP, Deep capillary plexus.

**Figure 3:**
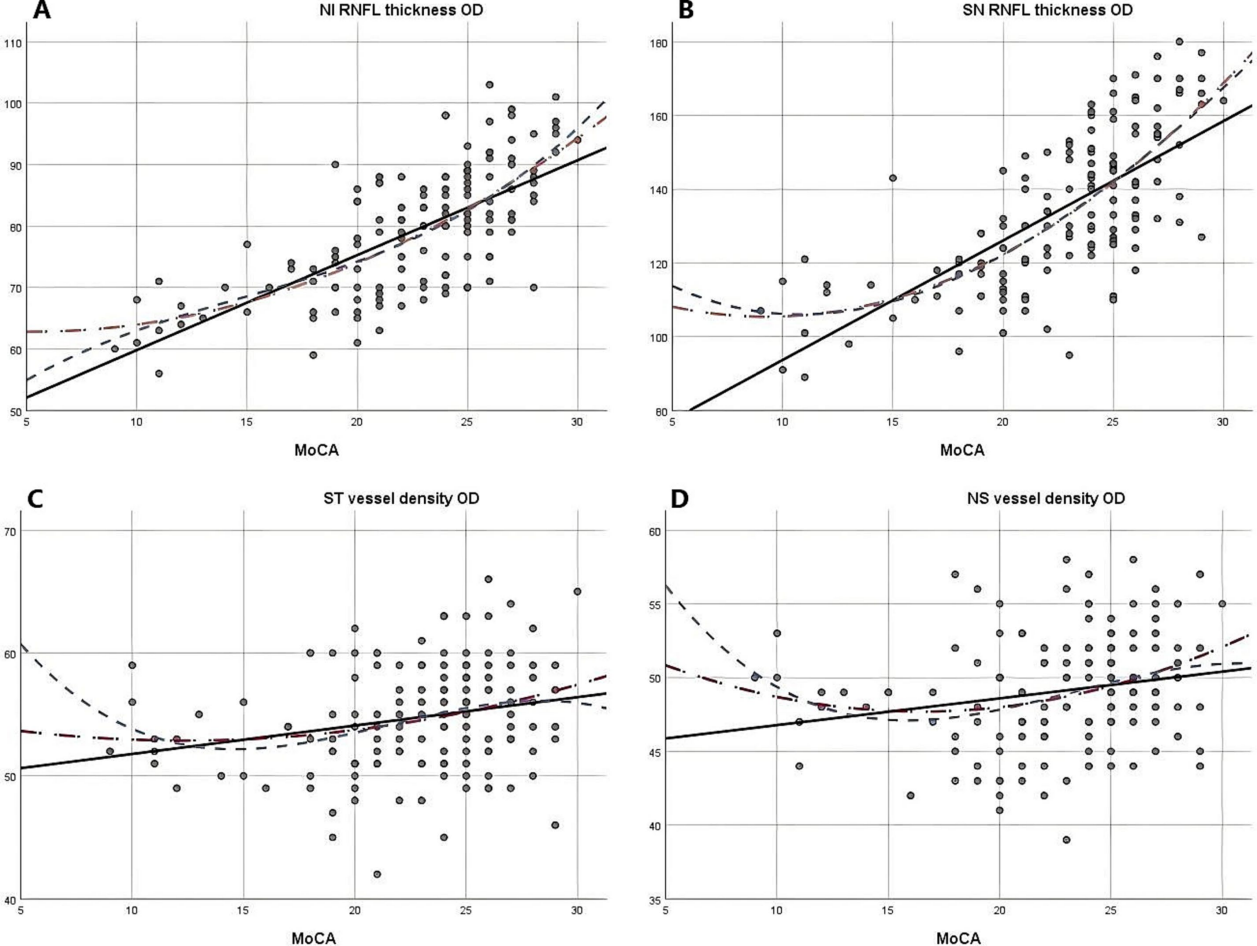
The relationship between Moca score and vascular density and RNFL thickness (SPSS Statistics 26 IBM); **A**: Moca score and right eye NI RNFL thickness; **B**: Moca score and right eye SN RNFL thickness; **C**: Moca score and right eye ST vascular density; **D**: Moca score and right eye NS vascular density; MoCA: Monterey Cognitive Assessment Scale; RNFL: Retinal nerve fiber layer.

**Figure 4:**
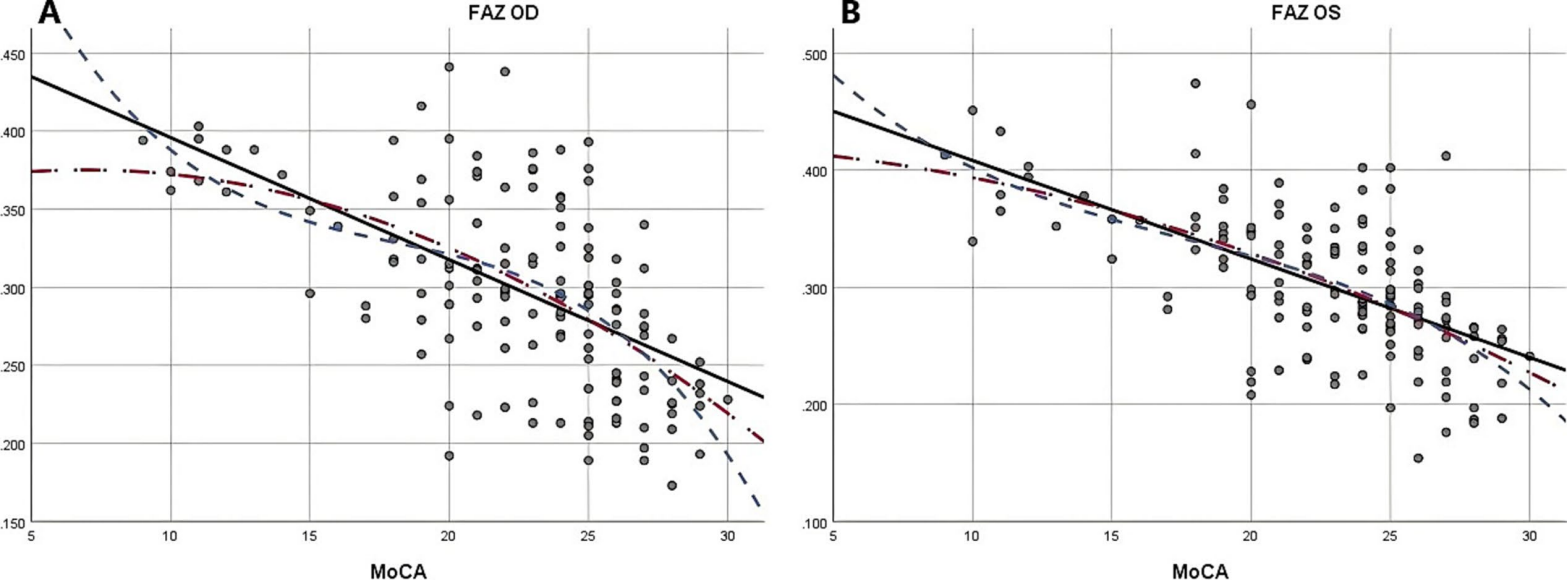
The relationship between Moca score and FAZ (SPSS Statistics 26 IBM); **A**: Moca score and right eye FAZ; **B**: Moca score and left eye FAZ; MoCA: Monterey Cognitive Assessment Scale; FAZ: Foveal avascular zone.

Due to the negative relation between WMLs burden, Fazekas scores, and cognitive function scores (22), MoCA scores were used to illustrate the linear relationship changes between WMLs severity and various OCTA parameters. In the quadratic simulated curve model and the cubic simulated curve model, MoCA scores were positively correlated with ILM-IPL thickness (Figure 1), parafoveal SCP and DCP in the macular area (Figure 2), and RNFL thickness at different locations (Figure 3). Each curve exhibits a monotonic relationship, lacking any evident threshold or inflection point. This indicates that as MoCA scores decrease, bilateral ILM-IPL thickness, parafoveal SCP/DCP vessel density, vessel density, and RNFL thickness decrease. Additionally, MoCA scores were negatively correlated with FAZ area, suggesting that as MoCA scores decrease, bilateral FAZ area increases gradually (Figure 4).

## 4 Discussion

**Figure 5:**
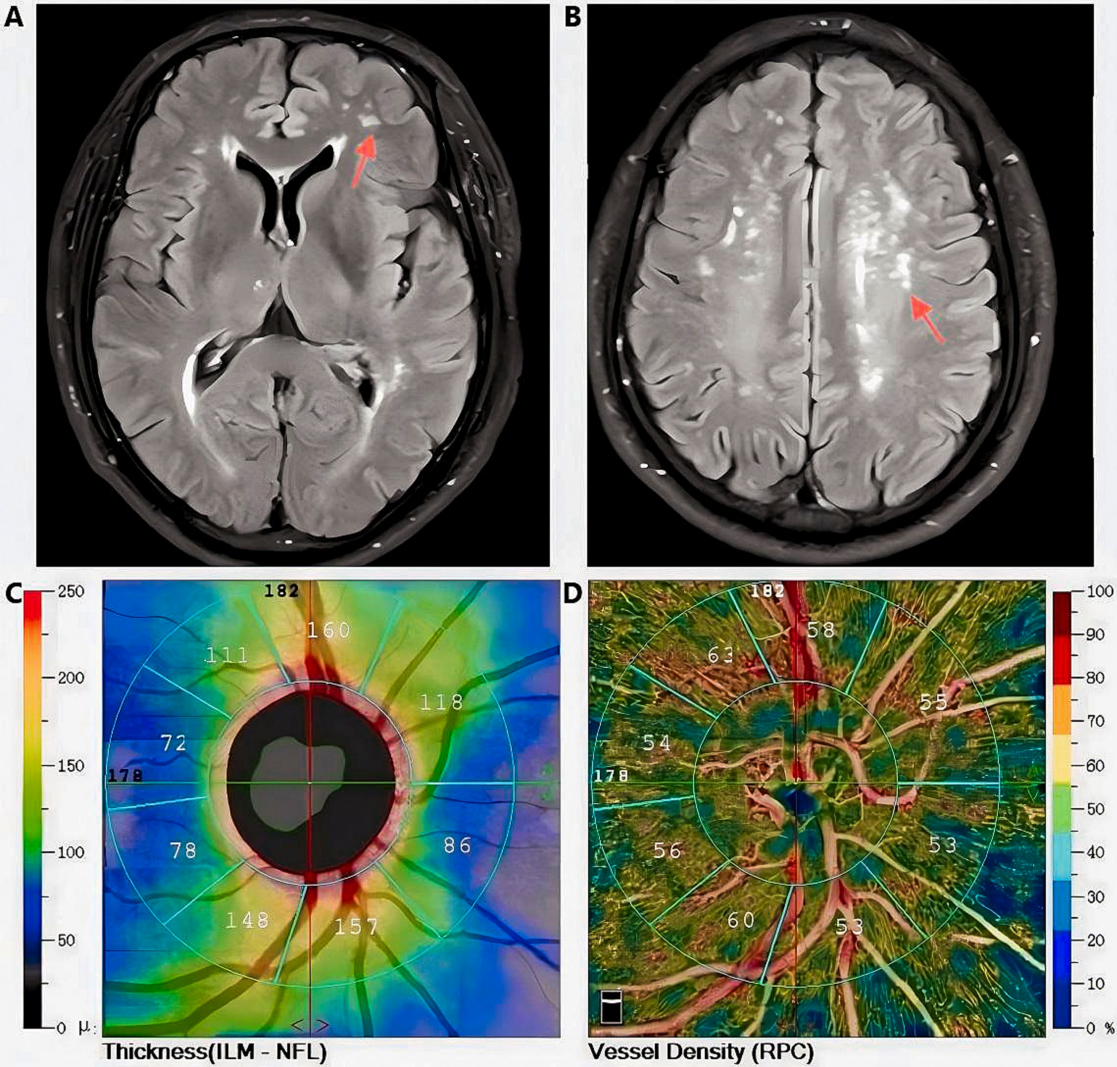
Magnetic Resonance Imaging T2-FLAIR characteristic images of white matter lesions (indicated by the red arrow) in a Fazekas grade 2 subject, along with retinal thickness and vascular density data obtained via Optical Coherence Tomography Angiography (Images from the same subject); **A, B**: Diagram of white matter lesions; **C**: Retinal thickness measurement diagram; **D**:Diagram of vascular density measurement.

This observational study systematically examined the relation between the extent of WMLs involvement and various retinal parameters, including ILM-IPL thickness, SCP and DCP vessel density, RNFL thickness, and FAZ area (Figure 5). The results showed that the degree of WMLs was significantly associated with various indicators of OCTA. As the white matter lesions progressively deteriorate, the vessel density and retinal thickness in all quadrants of the retina also decreased. Simultaneously, the FAZ region is progressively expanding. In other words, as the severity of white matter lesions increases, patients exhibit a thinner retinal thickness, reduced vascular density, and a larger extent of ischemia compared to healthy individuals.

The findings of this study demonstrate a significant relationship between the severity of WMLs in the brain and both retinal thickness and vascular status. The retina is intricately connected to the brain in both the nervous system and the vascular system. Based on our results, we suspect that wmls progression in the brain is accompanied by similar pathophysiological changes in the retina of patients. Previous research has indicated a correlation between elevated WMLs in the brain and alterations in both the retinal microvascular area and the avascular zone of the macula (18). These findings produce outcomes that are similar to those observed in our study. However, there’s also a study has found that changes in retinal nerve fiber layer thickness may not differentiate between normal individuals and those with preclinical Alzheimer’s disease (23). Additionally, OCT as a longitudinal screening tool for preclinical Alzheimer’s disease patients has limited efficacy (24). However, their data was based on OCT, and Alzheimer’s disease is a neurodegenerative disease that causes progressive neural functional degeneration. They did not consider cognitive decline caused by small vessel disease, which is frequently more prevalent and easier to develop than Alzheimer’s disease and has more complex risk factors, including blood pressure, blood sugar, smoking, and alcohol consumption.

In line with the previous studies, the odds ratios of participants with Fazekas grade 1 compared to grade 0 showed no significant differences in RNFL thickness and vessel density results in most quadrants (Except in the inferior nasal quadrant). However, significant differences were observed when comparing participants with Fazekas grades 2 and 3 to those with grade 0. As a result, it was concluded that RNFL thickness and certain areas of vessel density as observational indicators may have limitations in detecting early WMLs. Significant differences were observed in the inferior nasal RNFL thickness, superior nasal, temporal superior vessel density, and inferior nasal vessel density of participants with a grade of 0 compared to grade 1. In a study examining cognitive decline, the researchers proposed that in individuals without neurodegenerative diseases, a thinner RNFL was associated with poorer cognitive function and predicted more severe cognitive decline in the future (25). Comparable results were reported in a study, where they found that changes in retinal structure had a significant relation with cognitive function levels. Additionally, they showed that OCTA can detect early lesions. However, in contrast to our findings, they believed that vessel density in the superficial capillary plexus (inferior hemispheres, temporal and nasal quadrants) and deep capillary plexus (inferior hemispheres and nasal quadrants) of the macula may function as reliable biomarkers for detecting and monitoring early cognitive changes in Alzheimer’s disease (26).

Our new findings show similar characteristics in the FAZ area and the superficial capillary plexus beside the fovea. However, in the deep capillary plexus indicators, changes in the plexus more accurately reflected the progression of WMLs. Although multiple studies have reported a significant relation between retinal layer thickness and vascular changes with cognitive decline (27), they did not analyze the results subsequent to a comprehensive differentiation of the retinal regions.

Combining simulated curve analysis, we found that regardless of whether in the quadratic or cubic simulated curves, MoCA scores were positively correlated with ILM-IPL thickness, SCP and DCP in the parafoveal area, RFNL thickness in various locations and negatively correlated with FAZ area. Similar to the findings of a recent systematic review, this indicates that the extent of WMLs is negatively correlated with retinal thickness, vascular density, and other indicators and positively correlated with FAZ area (28). A study reported that thinner baseline total macular RNFL thickness was associated with a greater decline in MMSE scores during follow-up (29). However, detailed quadrant analysis was not performed on the total macular RNFL thickness at baseline in this study, and MMSE scores had a low sensitivity for patients with mild cognitive impairment.

In our simulated curves, we found that although the results of various indicators in different quadrants showed similar relations, they had different slopes, indicating varying degrees of response between retinal indicators and MoCA scores. This further confirms the challenge of certain indicators in detecting early WMLs. In particular, the inferior nasal quadrant of both eyes and the superior nasal quadrant of the right eye exhibited a steeper decline in RNFL thickness. However, contrary findings have been reported in some studies. Previous OCTA studies have demonstrated significant correlations between RNFL thickness, DCP, and the severity of WMLs when evaluated separately using the Fazekas scale (19). The RNFL, which contains retinal nerve axons reflecting the integrity of cerebral white matter (30), shows similarities to our findings. Our results thus reinforce the association between RNFL thickness and the pathogenesis of white matter hyperintensities. However, these earlier studies did not quantify RNFL thickness nor divide it into specific quadrants. Another study found that nasal RNFL thickness was significantly reduced in Alzheimer’s disease patients compared to those with hypercholesterolemia (31). These discrepancies may be due to different measurement methods or a lack of comprehensive quadrant analysis, which could introduce bias in the findings.

A comparable study conducted in China demonstrated that microvascular and axonal damage are associated with WMLs. The research indicated a significant impact on retinal blood vessel density, with these injuries correlating with disease severity and cognitive function. Furthermore, the study suggests that OCTA may serve as a valuable tool for quantifying retinal capillary density in patients with WMLs (19). A study compared Alzheimer’s disease patients to a normal control group and observed that all quantitative retinal nerve parameters (retinal nerve fiber layer thickness in the superior, inferior, nasal, and temporal quadrants) were decreased. Additionally, OCT scans detected a significant reduction in the thickness of macular RNFL during preclinical stages (32). Our curve results revealed a steeper slope in bilateral superior nasal, inferior nasal, and left nasal superior vascular density, which is consistent with the results of the aforementioned study. Furthermore, we provided a more comprehensive presentation of specific differences in various quadrants. We recently observed that the bilateral FAZ area and bilateral parafoveal SCP/DCP had significant slopes, with DCP showing a larger slope than SCP. Our findings provide new clinical evidence and directions for identifying patients with mild cognitive impairment in their early stages.

Recent OCT angiography studies have demonstrated that the FAZ area is significantly enlarged in patients with biomarker-positive cognitive dysfunction (33), suggesting that FAZ may serve as a potential biomarker in Alzheimer’s disease (34). Additional research has validated the findings of OCT angiography studies (35). These indicators exhibit a strong relation as a result of the close relationship between macular microvasculature changes (including FAZ area) and macular RNFL thickness. However, our study could not confirm whether the enlargement of the FAZ area preceded the thinning of macular RNFL thickness, which is the primary mechanism underlying the association between cognitive decline and changes in macular retinal microvasculature and neural structure (36).

In our preliminary research, we found that the occurrence and development of WMLs are likely correlated with local cerebral hypoperfusion (22). This condition is strongly associated with brain microvascular pathology, which can be attributed to various factors, including age and hypertension. In this study, it was observed that changes in retinal thickness and vascular density accompanied changes in the severity of WMLs. Due to their comparable anatomical and physiopathological characteristics, retinal and cerebral vessels are likely to exhibit similar processes and degrees of pathology development and share similar risk factors. Studies from Rotterdam have long suggested a relation between retinal venous dilation and the progression of cerebral microvascular disease, suggesting that the underlying mechanisms of vascular pathology between the retina and the brain are comparable (37).

Multiple studies have demonstrated that thicker retinal veins are correlated with higher cerebrospinal fluid pressure, and the increased ratio of retinal vein to artery diameter depends on elevated cerebrospinal fluid pressure, which is associated with blood pressure (38). Furthermore, age-related vascular changes have been identified as significant risk factors for both cognitive decline and small vessel disease (39). The retinal nerve fiber layer thickness and vessel diameter decrease with age as part of the normal aging process. However, these physiological changes may indicate degeneration in the brain’s white matter. Consistent with existing research, another study has demonstrated that alterations in retinal microvascular density, morphological parameters, and RNFL thickness are associated with the incidence of moderate-to-severe WMLs. These findings suggest that arteriosclerosis and hypoperfusion may underlie the pathogenesis of WMLs (40). Consequently, novel evidence suggesting concurrent changes in brain white matter may be discerned more easily through the utilization of OCTA to analyze alterations in retinal vessels and nerves.

There were several limitations to this study. First, it was a single-center observational study with a relatively small and homogenous sample size. Due to time constraints, the sample size for Fazekas 2 and 3 subjects was considerably smaller compared to the other two groups. This discrepancy may compromise the reliability and precision of both the logistic regression analysis and curve simulation outcomes. We collected data on multiple parameters of OCTA, which allowed for a more precise and comprehensive presentation of the data and findings. However, there are currently no universally recognized and standardized data criteria, and the risk of interference errors resulting from varying degrees of precision among measurers and different measuring instruments is significant. The MoCA score also has certain limitations. The test places high demands on the educational background of participants, and its assessment value for individuals with moderate to severe cognitive impairment is limited. This may result in scores that do not fully reflect the true cognitive function levels of the subjects.

## 5 Conclusion

This study established that the severity of white matter lesions increases as retinal thickness and vascular density decrease. Patients with poorer cognitive ability had thinner retinal thickness, lower fundus vessel density, and greater retinal ischemia. OCTA examination has a degree of role in screening for WMLs caused by cerebral microvascular disease. However, its screening effect on asymptomatic patients in the early stage is limited. Furthermore, there are differences in the response level of RNFL thickness and vascular density indicators in different retinal quadrants. Retinal indicators of OCTA in the bilateral superior-nasal, nasal-inferior, and left nasal-superior quadrants may be more effective clinical screening biomarkers for white matter lesions.

## Data Availability

The original contributions presented in the study are included in the article/Supplementary material, further inquiries can be directed to the corresponding authors.
