# Supplementary material for: Retinal assessment in 143 patients with white matter lesions: the potential of OCTA as an evaluation tool
Source: Front Neurol. 2025 Feb 6;16:1421232. doi: 10.3389/fneur.2025.1421232 (PMC11840757; doi:10.3389/fneur.2025.1421232)
Supplement: Supplementary file 1 [file Table_1.DOCX]

**Supplementary Table S1.** Multivariate logistic regression analysis of MRI-Fazekas grading and some indicators

| **Variables** | OD | | OS | |
| --- | --- | --- | --- | --- |
|  | OR (95% CI) | *P* value | OR（95%CI） | *P* value |
| TS RNFL thickness（F3） | 1.000 | - | 1.000 | - |
| F2 | 1.105（0.978 - 1.249） | 0.109 | 1.137（0.970 - 1.334） | 0.113 |
| F1 | 1.333（1.142 - 1.556） | ＜0.001 | 1.347（1.132 - 1.603） | 0.001 |
| F0 | 1.543（1.296 - 1.836） | ＜0.001 | 1.472（1.212 - 1.786） | ＜0.001 |
| SN RNFL thickness（F3） | 1.000 | - | 1.000 | - |
| F2 | 1.258（1.048 - 1.509） | 0.014 | 0.998（0.946 - 1.053） | 0.947 |
| F1 | 2.117（1.378 - 3.245） | 0.001 | 1.057（1.010 - 1.107） | 0.017 |
| F0 | 2.549（1.639 - 3.964） | ＜0.001 | 1.077（1.022 - 1.134） | 0.005 |
| NS RNFL thickness（F3） | 1.000 | - | 1.000 | - |
| F2 | 1.217（1.060 - 1.397） | 0.005 | 1.214（1.081 - 1.363） | 0.001 |
| F1 | 1.381（1.176 - 1.622） | ＜0.001 | 1.292（1.146 - 1.456） | ＜0.001 |
| F0 | 1.477（1.250 - 1.743） | ＜0.001 | 1.337（1.180 - 1.516） | ＜0.001 |
| NI RNFL thickness（F3） | 1.000 | - | 1.000 | - |
| F2 | 1.597（1.152 - 2.215） | 0.005 | 1.324（1.101 - 1.591） | 0.003 |
| F1 | 2.069（1.445 - 2.962） | ＜0.001 | 1.643（1318 - 2.047） | ＜0.001 |
| F0 | 2.799（1.885 - 4.155） | ＜0.001 | 2.083（1.619 - 2.681） | ＜0.001 |
| IT RNFL thickness（F3） | 1.000 | - | 1.000 | - |
| F2 | 1.049（0.982 - 1.119） | 0.156 | 1.071（0.978 - 1.173） | 0.139 |
| F1 | 1.170（1.080 - 1.267） | ＜0.001 | 1.254（1.124 - 1.399） | ＜0.001 |
| F0 | 1.200（1.103- 1.304） | ＜0.001 | 1.249（1.154 - 1.450） | ＜0.001 |
| TS vessel density（F3） | 1.000 | - | 1.000 | - |
| F2 | 1.540（1.212 - 1.957） | ＜0.001 | 1.612（1.204 - 2.518） | 0.001 |
| F1 | 1.615（1.266 - 2.059） | ＜0.001 | 1.755（1.309 - 2.353） | ＜0.001 |
| F0 | 1.997（1.525 - 2.616） | ＜0.001 | 2.041（1.468 - 2.839） | ＜0.001 |
| ST vessel density（F3） | 1.000 | - | 1.000 | - |
| F2 | 1.407（1.070 - 1.850） | 0.014 | 1.220（0.951 - 1.564） | 0.117 |
| F1 | 1.471（1.185 - 1.827） | ＜0.001 | 1.438（1.134 - 1.823） | 0.003 |
| F0 | 1.733（1.340 - 2.241） | ＜0.001 | 1.623（1.250 - 2.106） | ＜0.001 |
| SN vessel density（F3） | 1.000 | - | 1.000 | - |
| F2 | 1.266（0.962 - 1.665） | 0.092 | 1.052（0.823 - 1.344） | 0.686 |
| F1 | 2.112（1.475 - 3.024） | ＜0.001 | 1.541（1.183 - 2.008） | 0.001 |
| F0 | 2.520（1.718 - 3.697） | ＜0.001 | 1.632（1.216 - 2.189） | 0.001 |
| NI vessel density（F3） | 1.000 | - | 1.000 | - |
| F2 | 1.442（1.081 - 1.923） | 0.013 | 4.622（1.683 - 12.694） | 0.003 |
| F1 | 1.799（1.315 - 2.461） | ＜0.001 | 7.802（2.681 - 22.704） | ＜0.001 |
| F0 | 2.200（1.577 - 3.069） | ＜0.001 | 16.848（5.420 - 52.368） | ＜0.001 |
| IN vessel density（F3） | 1.000 | - | 1.000 | - |
| F2 | 1.138（0.920 - 1.408） | 0.235 | 1.108（0.857 - 1.432） | 0.433 |
| F1 | 1.217（1.009 - 1.469） | 0.040 | 1.274（0.996 - 1.629） | 0.053 |
| F0 | 1.388（1.107 - 1.740） | 0.005 | 1.618（1.218 - 2.147） | 0.001 |
| IT vessel density（F3） | 1.000 | - | 1.000 | - |
| F2 | 1.102（0.879 - 1.381） | 0.401 | 1.151（0.974 - 1.361） | 0.100 |
| F1 | 1.334（1.072 - 1.659） | 0.010 | 1.261（1.040 - 1.529） | 0.018 |
| F0 | 1.613（1.235 - 2.107） | ＜0.001 | 1.620（1.247 - 2.106） | ＜0.001 |

S: Superio; I: inferior; T: temporal; N: nasal；OD: Oculus dexter; OS: Oculus sinister
